# An anaerobic in vitro flow model for studying interactions at the gastrointestinal host–microbe interface

**DOI:** 10.1038/s41522-025-00800-z

**Published:** 2025-08-11

**Authors:** L. L. Bang, J. S. Pettersen, N. Høiland, A. M. Rojek, D. R. Tornby, J. Møller-Jensen, U. S. Justesen, R. M. Pedersen, T. E. Andersen

**Affiliations:** 1 Department of Clinical Microbiology, Odense University Hospital, Odense, Denmark; 2 Department of Clinical Research, University of Southern Denmark, Odense, Denmark; 3 Department of Biochemistry and Molecular Biology, University of Southern Denmark, Odense, Denmark; 4 Department of Pathology, Odense University Hospital, Odense, Denmark

**Keywords:** Clinical microbiology, Pathogens

## Abstract

In vitro research on host–microbe interactions in the human gut has been challenging due to the differing oxygen requirements of mammalian cells and intestinal microbiota. Few models of this environment have been developed, and those available are complex, limiting the extraction of important information during experiments. Here we report an in vitro model that by simple means creates an anaerobic environment for microbiota growing on living, cultured human epithelium under physiological flow. This model enables long-term co-culture of intestinal epithelial cells with obligate anaerobic bacteria, exemplified here by *Clostridioides difficile* and *Bacteroides fragilis*. Anaerobic conditions are maintained through the integration of an anaerobization unit, developed to facilitate online deoxygenation of media via liquid-to-liquid gas diffusion, eliminating the need for encapsulation in complex gas chambers. We show that stable oxygen levels of less than 1% can be maintained in the model for several days without compromising the viability of the intestinal epithelium. Furthermore, we demonstrate the performance of the model by simulating prolonged colonization with *C. difficile* and *B. fragilis*, as well as the clinically relevant persistence of *C. difficile* following treatment with vancomycin.

## Introduction

The human colon is a highly complex biological niche hosting trillions of bacteria that exist in symbiosis with the colon epithelium. This bacterial community, known as the microbiota, includes at least 500–1,000 different species, predominantly obligate anaerobes^1–4^. The microbiota plays a vital role in maintaining various aspects of human health^5^, including serving as a barrier to gastrointestinal pathogens^6^. Disruption or dysbiosis of the microbiota is a major risk factor for acquiring intestinal infections, such as *Clostridioides difficile* infection (CDI). Understanding the complex interaction between host, microbiota, and pathogen is critical for the development of effective treatment strategies. Until recently, researchers have primarily relied on animals as surrogate models of human intestinal infection^7^ and static microtiter plate cell culture models^8,9^, both of which have significant limitations. For example, traditional in vitro cell culture systems, such as cell culture microtiter plate inserts^8,9^, fail to mimic the complex conditions of the colon as they lack key in vivo-like features, such as the three-dimensional (3D) architecture of the intestinal epithelium, its anaerobic environment, and shear stress. Furthermore, these static systems can only be used for short-term studies. In contrast, animal models provide valuable insights into gut physiology, but they do not fully replicate the human gut environment or human susceptibility to infection. As a result, critical aspects of the pathogenesis of anaerobes remain poorly understood, complicating the development of treatment strategies. This is particularly the case for *C. difficile* infection, where the lack of qualified surrogate models recently led researchers to suggest developing a *C. difficile* infection model in humans^10^.

In recent years, intestine-on-a-chip systems have emerged as a promising platform for studying long-term co-cultures of bacteria and intestinal cells. The gold standard approach to manufacturing these chips is by soft lithography with polydimethylsiloxane (PDMS), a highly flexible, biocompatible material which is typically coated with extracellular matrix (ECM) for cell adhesion^11^. Indeed, PDMS chips have revolutionized the field of organ-on-chips, including models simulating the human intestine^12^. However, PDMS and other rubber materials compatible with soft lithography are also highly gas permeable, which compromises the chip’s compatibility with cultures that require strict separation and control of oxygen. Engineers have solved these problems in pilot setups by placing the chips in nitrogen containers^13^ or by shielding against oxygen leakage by various means^14,15^. A common characteristic of these solutions is that the systems have become complex. Combined with the expert knowledge required to produce and handle soft lithography chips, this has limited the dissemination of in vitro models to frontline clinical and industry researchers that develop solutions for infections and dysbiosis caused by anaerobic microorganisms^16^. The Caco-2 cell line, derived from a human colorectal tumor, is commonly used in these systems due to its ease of cultivation and proven barrier properties^17,18^. To mimic epithelial barrier characteristics, some systems incorporate parallel microchannels separated by a microporous membrane, enabling modeling of both the vascular and intestinal luminal environment within the same device. However, to simulate the hypoxic environment of the gut that enables growth of anaerobic bacteria, gases must be strictly separated to create the anaerobic environment on the intestinal luminal side, while providing oxygen to the intestinal cells from below. The latter feature has proven technically challenging and the few published models that have succeeded^19^ are highly complex setups that so far have not translated from laboratory pilots to more widely applicable systems.

To our knowledge, only three suggested solutions have been reported that support a 3D intestinal epithelium co-cultured with obligate anaerobic bacteria for several days. One model was published by Jalili-Firoozinezhad et al. in 2019 and consists of a PDMS chip lined with Caco-2 and endothelial cells, which allows for the culture of human microbiota for up to three days^13^. Hypoxic conditions (O_2_ < 2%, gas-phase equivalent) were maintained in the upper luminal channel by placing the chip in a box filled with nitrogen gas, while perfusing the lower vascular channel with oxygenated medium, to supply the intestinal cells with oxygen through the porous membrane. The authors demonstrated proof of replication of *Bacteroides fragilis* (*B. fragilis*) in the anaerobic chip and measured oxygen levels below 1% during the three-day culture period. Jalili- Firoozinezhad et al. further demonstrate the performance of the model by culturing primary ileal epithelium and gut microbiota. A second model, published by Shin et al. in 2019, likewise consisted of a modified PDMS chip but with a thicker upper part and a glass coverslip underneath the chip to control the oxygen flux through the gas-permeable PDMS. The authors computationally simulated the oxygen levels in the intestinal chip using measurements from experiments with a chemical oxygen scavenger in the medium. The authors estimated that the two obligate anaerobes *Bifidobacterium adolescentis* and *Eubacterium halili* remain viable for up to seven days, although no evidence of bacterial replication was provided^14^. In 2023, Liu et al. reported a soft lithography PDMS model where oxygen levels were also computer simulated and reoxygenation of the upper channel was prevented by attaching a glass coverslip to the outside of the chip^15^. Similarly, no evidence of bacterial replication and real-time oxygen measurements during co-culture were reported.

Common to the above models is the generation of anaerobic conditions in the chip by liquid-air passive diffusion of oxygen. This is however an equipment-intensive, slow process that requires encapsulation of the chip in sealed nitrogen containers that additionally limits the possibility to access the chip for material and data extraction.

In this study, we propose a novel approach to designing an in vitro flow model that enables the co-culture of intestinal cells and anaerobic bacteria. Instead of relying on oxygen permeable PDMS chips and the necessary encapsulation in anaerobic incubators, we use hard, oxygen impermeable flow chambers combined with a novel method for fast online anaerobization of media to the upper (intestinal lumen) channel.

The flow model supports co-culture with obligate anaerobic bacteria for at least five days, without compromising the viability of the cell layer. The model can be used to study host–microbe interactions and novel treatments against obligate anaerobic pathogens, for which in vitro models are urgently needed^20^. Using this model, we demonstrate, to our knowledge for the first time, sustained colonization by *C. difficile* on a live 3D intestinal epithelium over several days. Additionally, we monitored the bacterium’s response to vancomycin treatment in vitro, which reproduced the clinically challenging ability of *C. difficile* to survive standard antibiotic treatment in vivo. Contrary to earlier reported and commercial models, the reported concept is readily applicable in standard cell culture laboratories and generates highly stable co-cultures with even highly oxygen-sensitive bacteria.

## Results

### Establishment of the hypoxic intestinal lumen environment

**Fig. 1:**
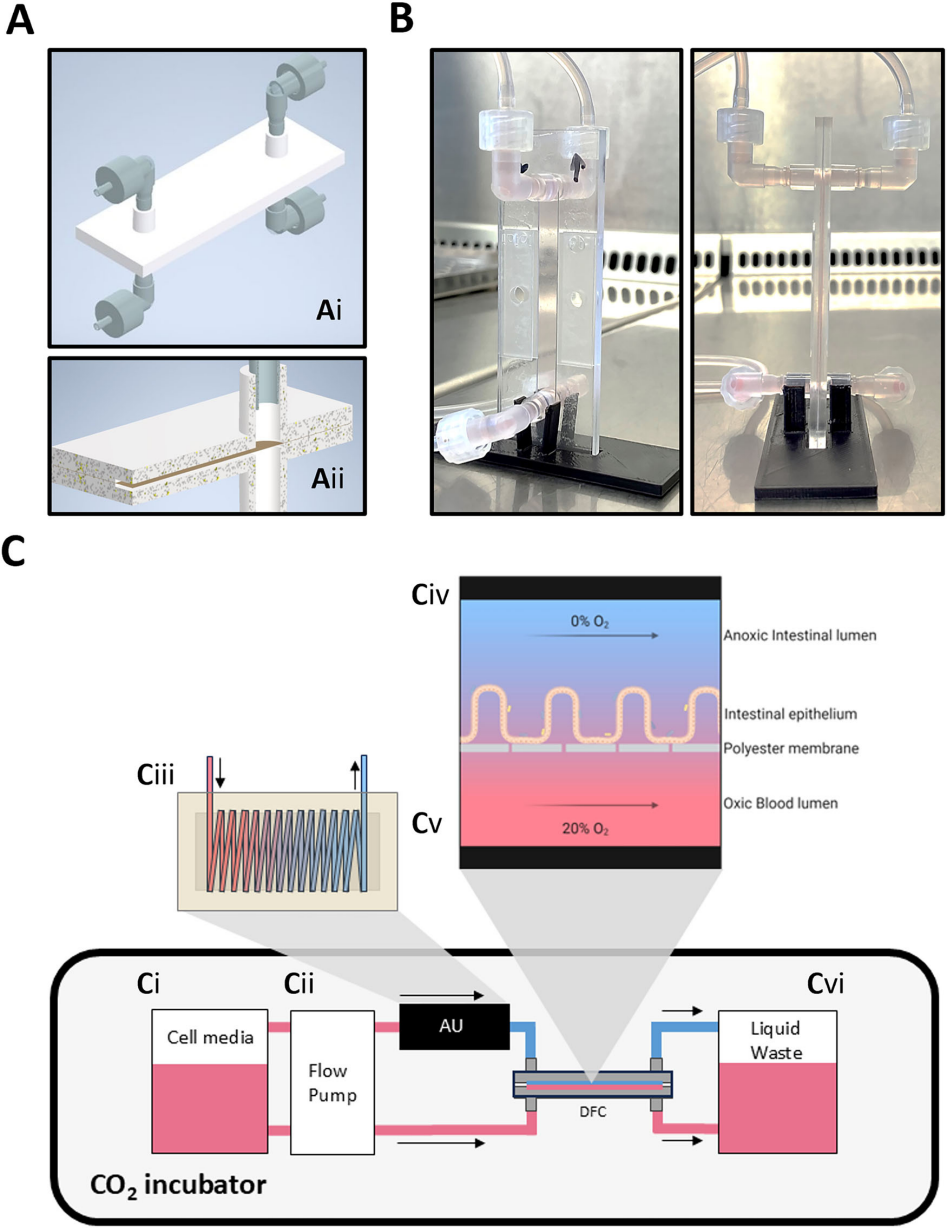
Design of the dual flow chamber and system setup. **A** Three-dimensional images of the dual flow chamber (DFC) showing the entire DFC with luer connectors (**Ai**) and a cross-section showing the microporous membrane in brown (**Aii**). **B** Real life images of the DFC during an aerobic experiment. The DFC dimensions without luer connectors are 75 × 25 × 4 mm. The DFC is placed in an upright position to prevent the accumulation of air bubbles. **C** Schematic presentation of the system setup placed in a standard CO_2_ incubator. Cell culture media (**Ci**) is pumped via the flow pump (**Cii**) through the anaerobization unit (AU) containing the silicone coil submerged in an antioxidant solution (**Ciii**) which is directly connected to the apical channel of the DFC (**Civ**) via a stainless-steel tube. Aerobic cell media is supplied through the basolateral channel (**Cv**) using the same flow pump (**Cii**). Cell media exiting the DFC is collected in the liquid waste container (**Cvi**). Figure was made with Biorender.com and Microsoft Powerpoint.

**Fig. 2:**
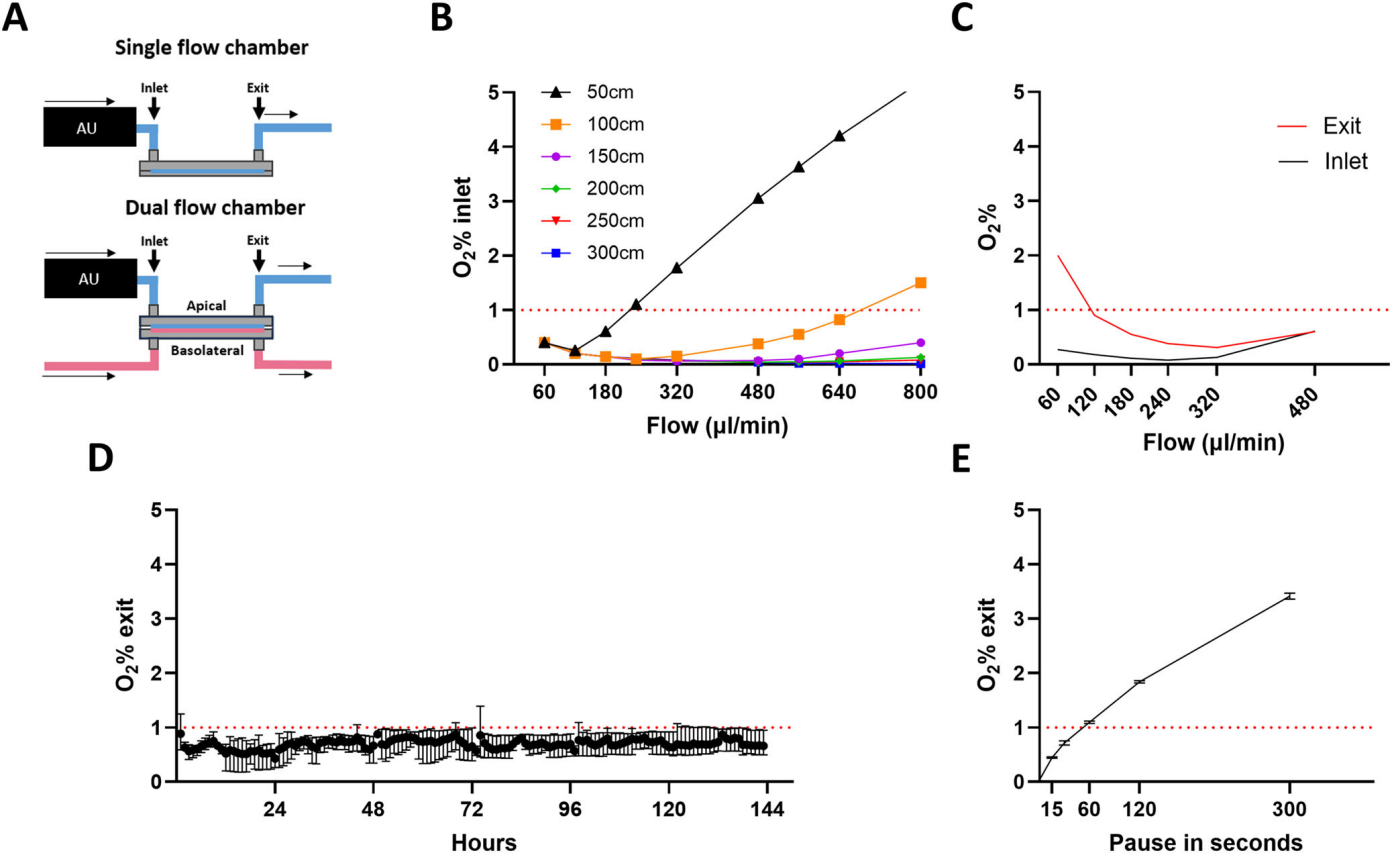
Oxygen levels and dependency on flow rate, tube length and chamber conditions. **A** Schematic presentation of the single and the dual flow chamber (DFC) showing the connection sites of the oxygen sensor (indicated "Inlet" and “Exit"). Inlet measurements were performed by connecting the oxygen sensor to the exit site of the anaerobization unit (AU) and exit measurements by connecting the oxygen sensor to the exit of the flow chambers. The AU and the flow chambers are connected with stainless-steel tubing to prevent reabsorption of oxygen into the cell media. **B** Inlet oxygen percentage measured using different lengths of the AU silicone tube coil and at different flow rates. **C** Oxygen percentage at inlet and exit sites in a single flow chamber (µ-slide I luer 0.4mm, Ibidi) at different flow speeds, showing the relationship between oxygen percentage and flow rate. **D** Oxygen percentage in effluent from DFCs with Caco-2 cells cultured under anaerobic conditions for 6 days with a flow of 320 µl/min. The data is based on an hourly average measured in three individual flow chambers (Mean ±range). Exit measurements represent the highest oxygen levels in the apical channel, as oxygen is slowly reabsorbed into the medium during passage through the channel. **E** Reabsorption of oxygen into the apical flow channel of a dual flow chamber, when the flow is stopped for different periods of time.

To establish a model closely simulating the physiology and microenvironment of the human colon, we aimed to create a system that incorporates the shear stress conditions and the low-oxygen environment present in the colon, while supporting the growth and maintenance of an intestinal epithelial cell culture. We chose the dual flow channel principle for the model. Contrary to PDMS dual-channel chips, however, in which media gas content is controlled by the gas outside of the chip due to the gas-permeable PDMS, we opted for a hard-plastic system that would eliminate the need for encapsulation in gas containers (Fig. 1A, B). We intended to establish such a stand-alone system that would be compatible with physiological wall shear stress of at least 0.1 dyn/cm^2^ and media oxygen levels below 1%, both of which approach the conditions in the intestine^21–23^. Realizing such a system required a novel method for fast and effective online deoxygenation of flow media, before reaching the chamber. Our solution exploits the fast diffusion of oxygen through silicone rubber and the highly oxygen-attractant properties of antioxidant liquids^24^. An ultrathin silicone tube is coiled within a container filled with a strong aqueous antioxidant solution (Fig. 1C). Passing liquid media through this system rapidly depletes dissolved oxygen, even at high flow rates (Fig. 2). The dual flow chamber (DFC) was made by mounting two Ibidi® sticky slides® back-to-back with a thin, porous, transparent (track-etched) polyester membrane in between (Fig. 1A, B, Supplementary Fig. S1). This design created apical and basolateral flow channels within the DFC, with the outer walls acting as effective oxygen barriers, while oxygen readily diffuses across the culture membrane from the lower channel to the intestinal cell culture. Oxygen concentrations were measured in the media exiting the anaerobization unit (AU) to test the deoxygenation efficiency, and in the media exiting single- and DFCs to monitor reabsorption of oxygen during flow chamber passage (Fig. 2A–C, E). As demonstrated in Fig. 2, a rate of oxygen depletion was achieved that allowed flow rates necessary for simulating the physiological liquid shear of the intestine. Several factors influence the deoxygenation efficiency, including media flow rate and tube wall thickness, length, and diameter (Fig. 2B, C, E).

To provide deoxygenized media at flow rates of 120–640 µl/min, necessary to generate the desired 0.1–0.6 dyn/cm² in DFCs, a tube with luminal diameter of 0.99 mm, wall thickness of 0.31 mm, and length of ≥150 cm was used in the AU (Fig. 2B).

To avoid variations due to the consumption of oxygen by the living epithelium, oxygen reabsorption kinetics in media during channel passage was measured in a single-channel flow chamber without cells, with channel dimensions matching the DFC’s apical channel (Fig. 2A). The results indicated a flow rate of 320 µl/min (shear stress of 0.3 dyn/cm²) as a sweet spot with both low oxygen levels and little difference between exit and inlet measurements (Fig. 2C). Measurements of oxygen levels passing through DFCs with 7-days matured Caco-2 epithelium confirmed consistently low anaerobic conditions over the following six-days, reaching levels between 0.1-1% (Fig. 2D). During flow interruptions, oxygen concentration measurements revealed that flow could be paused for up to 60 s before exit levels exceeded 1%, and up to 5 min before surpassing 3% (Fig. 2E). Oxygen levels decreased to <1% within a few minutes after resuming the flow.

### Caco-2 cells matured in the DFC demonstrate in vivo characteristics of the intestine

**Fig. 3:**
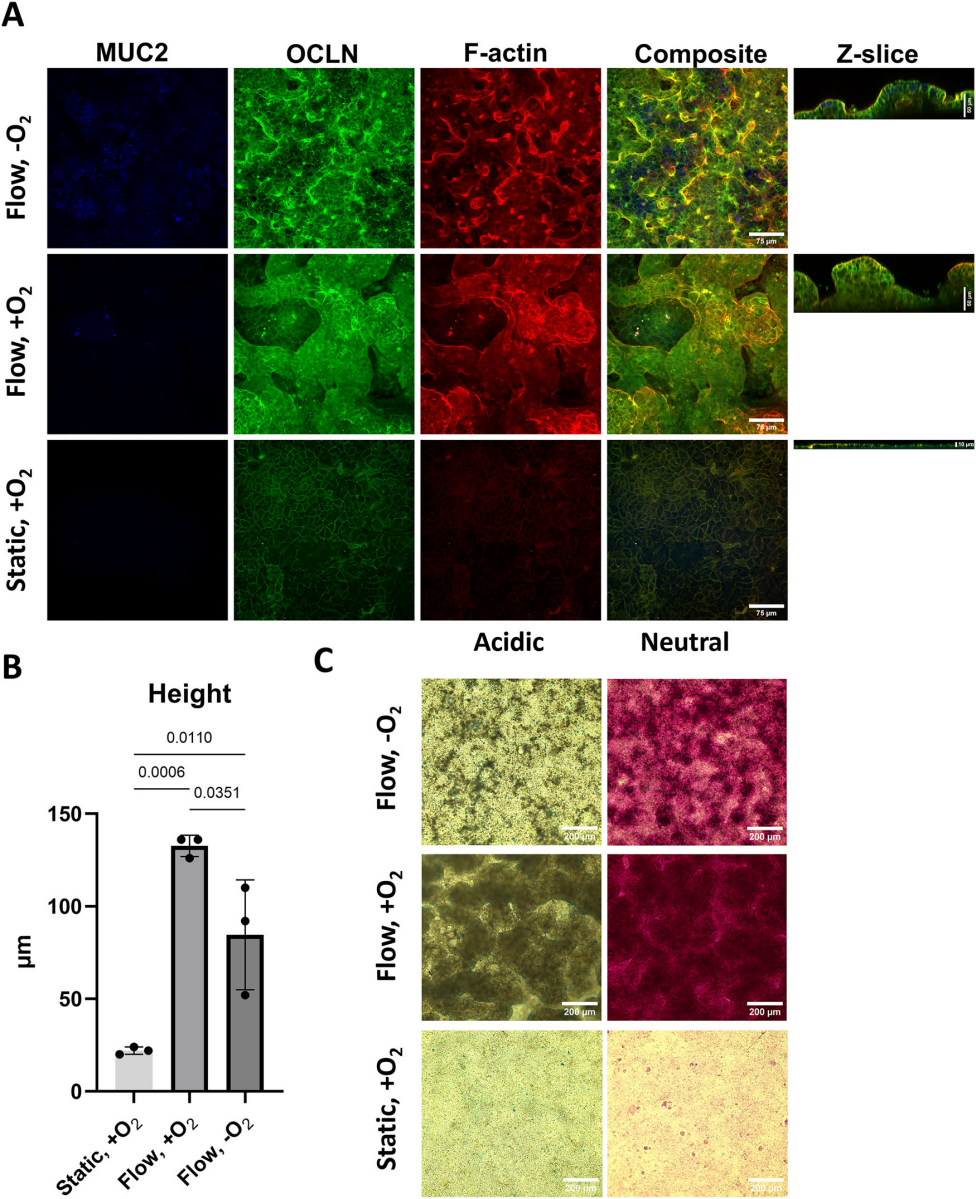
Characterization of the cultured Caco-2 epithelium 13 days post-seeding. **A** Representative confocal laser scanning microscopy z-stack images of Caco-2 cells grown under static (cell culture inserts) or flow conditions (dual flow chamber), showing the three-dimensional structure of the cultured epithelium. The anaerobization unit was connected to the apical inlet on day 7 post-seeding to create an oxygen gradient throughout the cell layer (−O_2_) and compared with experiments where both channels were kept aerobic (+O_2_). Cells were stained for Mucin 2 (MUC2), the tight junction protein Occludin (OCLN) and F-actin. Z-slices indicate cross sections based on z-stacks. Images are representative of two individual experiments with three images of each condition. **B** Heigh in micrometers (µm) of the cultured Caco-2 epithelium grown under the three different conditions. The height was determined based on confocal microscopy z-stacks. Error bars indicate standard deviation. Statistics were made using a one-way ANOVA with multiple comparisons. **C** Staining of neutral (Shiff’s reagent) and acidic (Alcian blue) mucins. For mucin expression at earlier time points, see Supplementary Fig. S3. Three representative pictures were taken for each condition.

For the first iteration of the model, we selected the human epithelial cell line Caco-2, a gold standard in many gut-on-a-chip models. After confirming that the model met the requirements for shear stress (SS) and oxygen levels, the characteristics of the Caco-2 cell layer cultured in the DFC under both aerobic and anaerobic conditions were investigated and compared to static cultures in cell culture inserts. Caco-2 cells cultured for 13 days in the DFC, formed a complex 3D epithelium with crypt- and villus-like structures, reaching up to 135 µm in height (Fig. 3A, B). Under anaerobic conditions in the apical channel, a greater variation in the height of the cell layer ranging between 50-115 µm was observed (Fig. 3B, Supplementary Fig. S2). By comparison, the average height of the static cultures after 13 days was approximately 20 µm (Fig. 3B). Under both aerobic and anaerobic flow, the mature Caco-2 layer stained positive for the tight junction protein occludin and the cytoskeleton component F-actin, indicating establishment of an intestinal barrier (Fig. 3A). To assess mucus production, the cultured tissue was stained for neutral and acidic mucins, and immunostained for MUC2, the primary gel-forming mucin in the colon. Both aerobic and anaerobic flow conditions revealed a higher signal of neutral mucins compared to the static control (Fig. 3C). Due to the height of the cell layer, it was difficult to make firm conclusions on the presence of acidic mucins, but some blue stains did appear under all three culture conditions (Fig. 3C, Supplementary Fig. S3). The presence of MUC2 by immunostaining was detected in the Caco-2 cells cultured under aerobic and anaerobic conditions in the DFC. MUC2 appeared mostly in vesicles within some of the cells at the top of the cultured epithelium. The low signal indicated minimal MUC2 production by the cells. Subsequent ELISA analysis confirmed this, showing MUC2 levels below the lower detection limit (0.78 ng/mL, data not shown). The Caco-2 epithelium was found to maintain viability for at least 21 days under anaerobic conditions (Supplementary Fig. S4).

### Transcriptomic profiling reveals enterocyte maturation in the Caco-2 DFC model

To comprehensively investigate the transcriptional profile of the intestinal epithelium cultured in the system, we conducted transcriptomic analyses on Caco-2 cells cultured for 13 days under both aerobic and anaerobic conditions in the apical channel of the DFC model, and statically under aerobic conditions in cell culture inserts. Differentially expressed genes (DEGs) were identified and analyzed by pathway enrichment analysis using Metascape^25^.

**Fig. 4:**
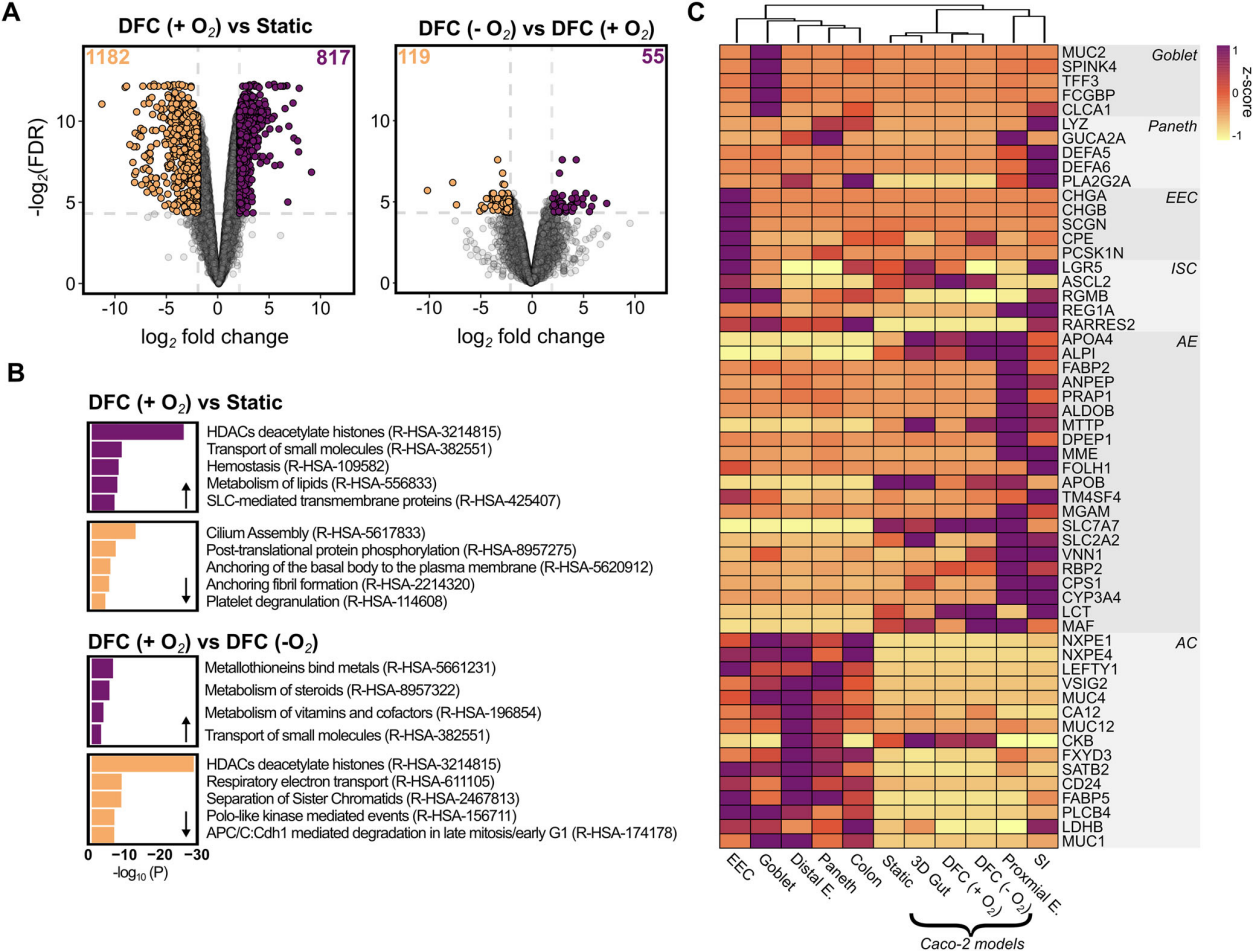
Transcriptomic characterization of the Caco-2-based DFC model. Caco-2 cells were cultured for 13 days in the DFC model, both aerobically (DFC + O_2_) and anaerobically (DFC-O_2_), as well as in cell culture inserts under aerobic conditions (Static), all in triplicates. RNA sequencing was then performed to analyze gene expression profiles. **A** Differentially expressed genes (DEGs) between Caco-2 cells cultured in the aerobic DFC and the static cell culture inserts, as well as between cells cultured in the anaerobic and aerobic DFC models. **B** Pathway enrichment analysis of Reactome gene sets for up- and downregulated genes in the aerobic DFC model compared to the cell culture inserts, and in the anaerobic DFC model compared to the aerobic DFC model. Here, top 5 enriched pathways are shown, unless fewer pathways were significantly enriched. **C** Clustered heatmap showing scaled marker gene expression profiles of six major intestinal cell types: goblet cells, paneth cells, enteroendocrine cells (EEC), intestinal stem cells (ISC), absorptive enterocytes (AE), and absorptive coloncytes (AC). This comparison includes data from Caco-2 models of this study, a Caco-2-based 3D gut model from Cheng et al.^22^ and single-cell and bulk RNA sequencing data of intestinal cell types (goblet, paneth, EEC, distal enterocytes, and proximal enterocytes) and tissues (colon and small intestine (SI)) derived from the Human Protein Atlas^57^.

Among the 817 upregulated genes in the aerobic DFC model relative to the static model, several were associated with the ‘HDACs deacetylate histones’ Reactome pathway, including multiple histone genes (Fig. 4A, B). Upregulation of histone genes, a marker of entry into the S phase of the cell cycle^26^, may indicate an increase in the number of actively dividing cells in the aerobic DFC, aligning with the observed increase in 3D growth compared to the static model. Additionally, pathways related to small molecule transport (R-HSA-382551 and R-HSA-425407) and lipid metabolism (R-HSA-556833) were enriched, pointing to enhanced absorptive function of the enterocytes. Conversely, among the 1182 downregulated genes, pathways related to cilia development and function (R-HSA-5617833 and R-HSA-5620912) were significantly enriched. Cilia, membrane-bound sensory organelles that detect signals such as shear stress, are typically present in quiescent cells and are generally absent in rapidly renewing tissues, including the intestinal epithelium^27^.

By contrast, only 174 genes were differentially expressed between the anaerobic and aerobic DFC models. The subtle difference in transcriptional activity suggests that the Caco-2 cells are provided adequate amounts of oxygen for sustaining a vital state of growth, despite the only oxygen source is the media flowing beneath the culture (Fig. 4A). Still, the upregulation of several metallothionein-encoding genes (R-HSA-5661231), which are involved in metal homeostasis and protection against oxidative stress, alongside the downregulation of genes related to the electron transport chain (R-HSA-611105), indicates a hypoxic response. Interestingly, significant transcriptional induction of hypoxia-inducible factor-1 (*HIF1A*) mRNA was not observed, although previous studies have reported increased HIF-1α protein levels under hypoxic conditions^13^ (Supplementary Fig. S5). Such a response is widely recognized as being vital for maintaining intestinal barrier integrity and regulating nutrient absorption within the intestinal epithelium^28^. Additionally, several pathways related to mitosis (R-HSA-3214815, R-HSA-2467813, R-HSA-156711, and R-HSA-174178) were also enriched among the downregulated genes, indicating a decrease in cell proliferation compared to the aerobic DFC model (Fig. 4B).

Although derived from the colon, Caco-2 monolayers exert many of the same properties as absorptive enterocytes of the small intestine^29^. To determine whether DFC conditions promote maturation toward a

more differentiated intestinal epithelium, their transcriptomic profiles were compared to various intestinal cell types and tissues. To determine the cell type resemblance, we analyzed marker gene expression for six major intestinal cell types: goblet cells, Paneth cells, enteroendocrine cells (EEC), intestinal stem cells (ISC), absorptive enterocytes (AE), and absorptive colonocytes (AC). Our data was compared with single-cell RNA-sequencing and bulk RNA-sequencing datasets, as well as a recently described Caco-2-based anaerobic 3D gut model^30^. The clustered heatmap (Fig. 4C) reveals that all the Caco-2 models most closely resemble (proximal) enterocytes of the small intestine, as reported in the literature^29,31^. Notably, the anaerobic DFC model exhibited higher expression of AE-specific markers (*APOA4*, *ALPI*, *MTTP*, *RBP2*, *MAF*, and *LCT*) compared to the static model, indicating a stronger AE profile. Expression levels of commonly reported genes in intestinal models, including VIL1 and genes associated with epithelial barrier integrity (TJP1, OCLN), were also within the range of in vivo human intestinal transcriptional profiles (Supplementary Fig. S5). Low expression of MUC2, previously demonstrated by immunostaining and ELISA, was confirmed at the transcriptional level, consistent with a small intestinal enterocyte phenotype and in line with data from the Caco-2-based anaerobic chip model described by Cheng et al. Overall, the transcriptional profile of our anaerobic DFC model closely mirrors that of the model by Cheng et al., demonstrating that a similar Caco-2 culture is achieved in our DFC model as in the more complex, anaerobic PDMS microfluidic-based systems.

### Co-culture of obligate anaerobes and Caco-2 cells in the DFC

**Fig. 5:**
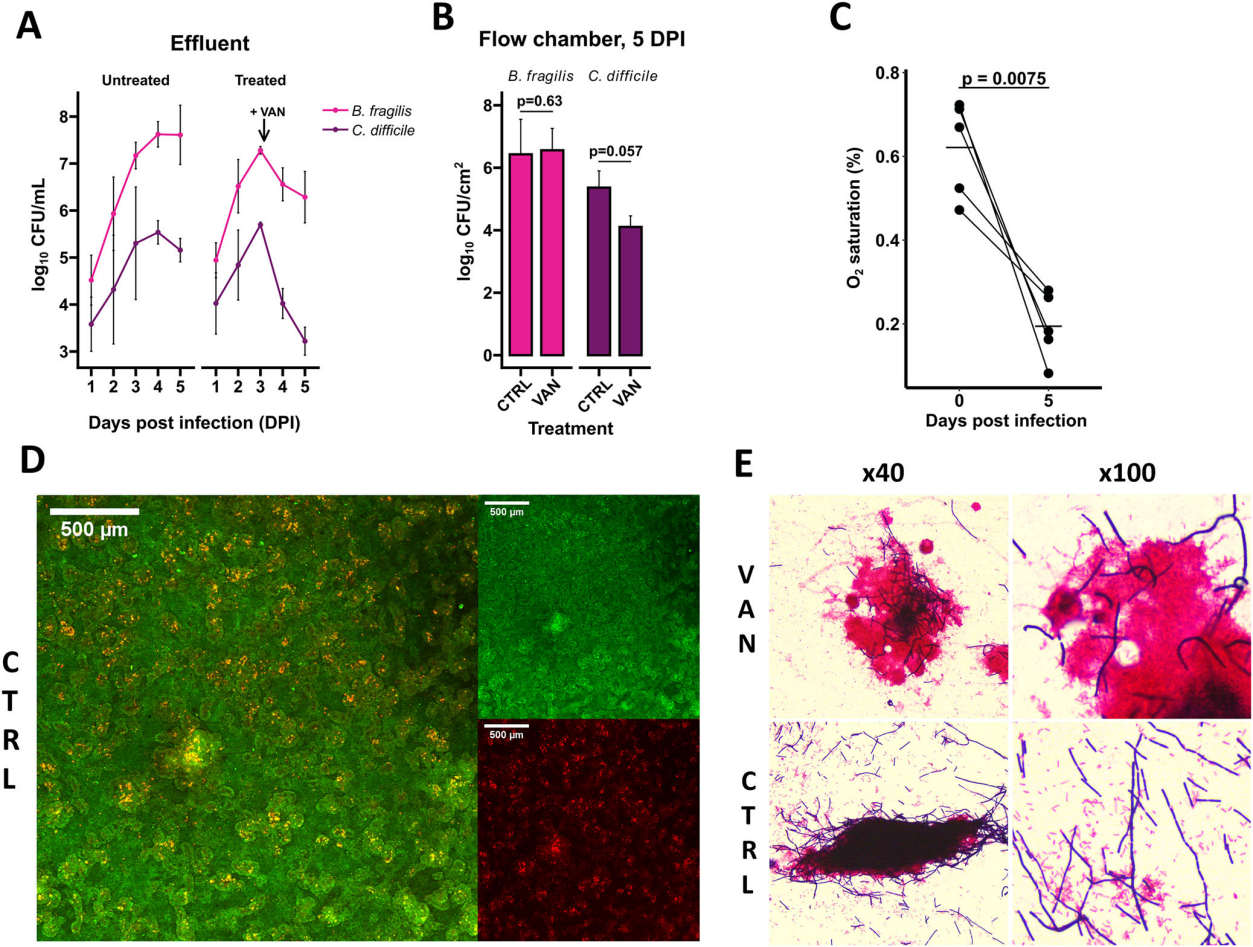
Colonization with *C. difficile* and *B. fragilis.* The cultured Caco-2 cell epithelium was infected with *C. difficile* and *B. fragilis* 7 days post-seeding in the dual flow chamber (DFC). On the third day post-infection (DPI), the bacteria-colonized epithelia were treated with 6 µg/mL vancomycin (VAN) or left untreated (CTRL). **A** Bacteria shed from the simulated infection is monitored in CFU/mL measured in the effluent from the apical channel of the DFC before and after vancomycin treatment. Data represent the mean ± standard deviation (SD) from three biological replicates. **B** Bacteria associated with the colonized epithelium harvested 5 DPI. The numbers of viable bacteria are shown in log_10_ CFU per cm^2^ of the epithelium. Data represent the mean ± standard deviation (SD) from three biological replicates. Statistical comparisons were made using an unpaired Wilcoxon test. **C** Oxygen levels (% O_2_, gas-phase equivalent) was measured at the DFC apical channel exit site before and at 5 DPI. Black lines indicate mean values. Statistical comparisons were made using a paired *t* test. **D** Confocal laser scanning microscopy images of LIVE/DEAD stained epithelium 5 DPI. Live cells stain green, dead cells stain red. The images are combined from z-stacks. Images are based on one experiment. **E** Gram stain of effluent containing shed bacterial clumps from the apical channel of the DFC 5 DPI. The gram-positive *C. difficile* stains blue while the gram-negative *B. fragilis* stains red.

To test if the model supports growth of obligate anaerobes, it was used to simulate colonization with the obligate anaerobic bacterial species *C. difficile*. The obligate anaerobe *B. fragilis*, a prominent member of the normal microbiota, was included to represent the commensal population^32–34^. Bacteria were inoculated seven days post-seeding of the Caco-2 cells in the DFC. After inoculation, bacterial colonization was monitored both macro- and microscopically and by plating the effluent from the apical channel daily. Three days post inoculation, areas resembling biofilm were visible to the naked eye, and the bacterial counts in the effluent exceeded 10^7^ CFU/ml for *B. fragilis* and 10^5^ CFU/ml for *C. difficile*, demonstrating that the system provides favorable growth conditions for these obligate anaerobic microorganisms (Fig. 5A, Supplementary Fig. S6). Following bacterial colonization, the viability of the intestinal epithelium was examined by viability staining and confocal laser scanning microscopy (CLSM). This analysis showed that a confluent layer of viable Caco-2 cells was present after the 5-day colonization with *C. difficile* and *B. fragilis* (Fig. 5D).

### Vancomycin treatment of the *C. difficile* colonized epithelium

To assess the DFC model’s applicability for testing antimicrobial treatment regimens, vancomycin treatment experiments were conducted. Vancomycin, a common antibiotic used to treat *C. difficile* infections, is effective against *C. difficile*, while *B. fragilis* tolerates the drug^34,35^. Treatment with vancomycin (6 ug/ml) was initiated three days post-infection (DPI). After two days of treatment, the CFU count of *C. difficile* in the effluent had reduced approximately 300-fold (Fig. 5A), but some bacteria were still present in the aggregates harvested from the chamber (Fig. 5E). Interestingly, a 10-fold reduction in the amount of *B*. *fragilis* was observed in the effluent, compared to the untreated control (Fig. 5A). However, when harvesting the cell layer to enumerate the sessile population, the same amount of *B. fragilis* was present in the vancomycin treated DFC, compared to the untreated control (Fig. 5B). The amount of *C. difficile* associated with the cell layer was reduced nearly 100-fold following vancomycin treatment, compared to the control (Fig. 5B). Despite this, high numbers of *C. difficile* were still present in the vancomycin treated DFC ( > 10,000 CFU/cm2). After treatment, the majority of *C. difficile* cells were observed in close association with *B. fragilis* aggregates, as indicated in Gram stains of supernatant samples collected from the apical channel of DFC (Fig. 5E). Measurements of the oxygen concentration at the apical channel exit before and after the five-day experiment showed a mean decrease in oxygen levels from 0.6 to 0.2% (Fig. 5C). This result shows that the barrier properties to oxygen remains intact and indicates an uncompromised epithelium despite bacterial colonization.

### Invasion of the intestinal epithelium

**Fig. 6:**
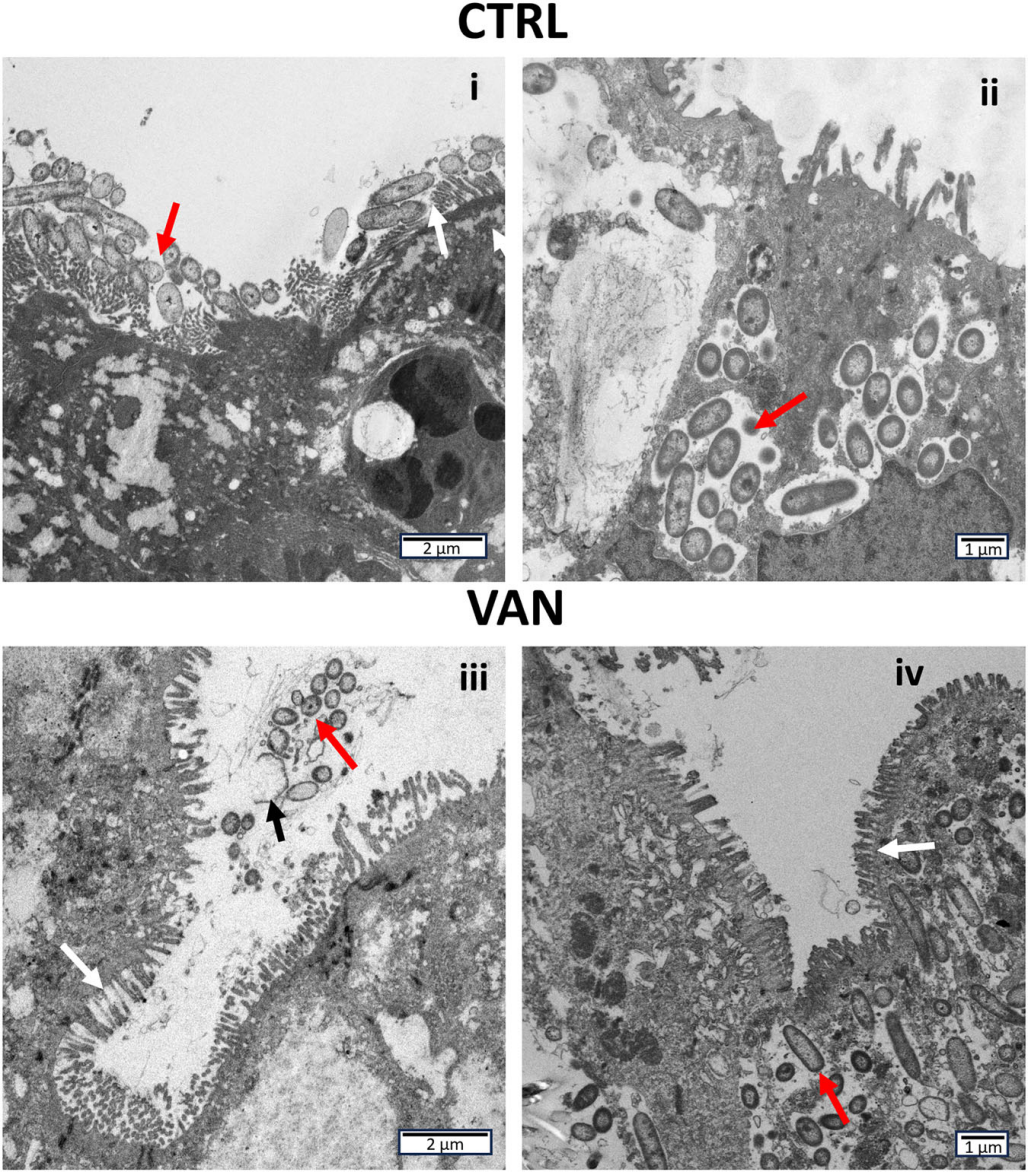
Transmission electron microscopy of bacterial colonization of cultured Caco-2 epithelium. Caco-2 epithelia cultured for 7 days in dual flow chambers (DFCs) were inoculated with *C. difficile* and *B. fragilis* and at 3 days post-infection (DPI) treated with 6 µg/ml vancomycin (VAN) or left untreated (CTRL). Bacteria and cultured Caco-2 epithelium were harvested 5 DPI. Bacteria were found both extra- (**i, iii**) and intracellular (**ii, iv**) in both the vancomycin-treated (**iii, iv**) and untreated (**i, ii**) DFCs. Red arrows indicate areas with colonizing bacteria. White arrows indicate microvilli on the surface of the Caco-2 cells. The black arrow indicates lysed bacteria.

To further explore the *C. difficile* and *B. fragilis* intestinal colonization strategy and assess the effect of vancomycin treatment, transmission electron microscopy (TEM) was performed on treated and non-treated, colonized epithelia. In the non-treated control, TEM revealed numerous bacteria located both extra- and intracellularly (Fig. 6, i and ii). In the vancomycin-treated bacterial co-culture, only a few bacteria were observed extracellularly, but numerous intracellular bacteria were observed by TEM (Fig. 6, iii and iv). The extracellular colonies contained what appeared to be lysed bacteria (Fig. 6, iii). Additionally, TEM analysis revealed a consistent layer of microvilli on the lumen of the cultured epithelium at 5 days post-infection (Fig. 6, iii and iv).

## Discussion

In this study, we present a novel in vitro model that allows for the co-culture of intestinal epithelium with obligate anaerobic bacteria for several days. The model is suitable for a variety of applications, such as studying host–bacteria interactions and preclinical testing of antimicrobial agents and can be used for simulating anaerobic environmental biofilms as well. Anaerobic conditions (<1% O_2_) can be maintained in the apical channel in principle indefinitely as it depends on an effective online oxygen depletion method specifically invented for the model^24^. The approach eliminates the need for time-consuming pre-anaerobization of media, encapsulation in N_2_-chambers, and breaking of seals when inspecting the chip or replacing media. The dual flow chamber (DFC) is oxygen-tight meaning that the DFC and attached anaerobization unit (AU) can be placed and handled in the open environment without risking oxygen leakage into the system, and the DFC culture can be maintained in a standard CO_2_ incubator. Furthermore, the AU can be individually adapted to fit the desired oxygen levels in other parts of the gastrointestinal tract or for simulating limited oxygen environmental biofilms, e.g. by shortening the silicone tube running through the AU, as demonstrated in this study.

The Caco-2 cell line is able to differentiate when exposed to liquid shear stress (SS), creating both villus-like structures and in some cases detectable mucus^36–38^, making it an excellent choice for establishing intestinal models of the small intestine. In the DFC, Caco-2 cells can be cultured for at least 21 days during which the cells differentiate to an epithelium with gut-like structures including villi, microvilli, crypts and mucus. The morphology and differentiation of Caco-2 cells cultured under low SS is well studied^39^ as gut-on-chip models often use a shear rate of 0.02 dyn/cm^2^ ^13–15^. However, it can be argued that this is significantly lower than the physiological SS present in the gut. One study has reported SS values in the small intestine of guinea pigs of up to 1.2 dyn/cm^2^ ^21^, while others suggest that the shear stress of the small intestine reaches levels between 0.1-10 dyn/cm^2^ during peristaltic movements^22^. However, the true SS of the human colon is yet to be explored. Only a few studies have characterized intestinal cells cultured under these higher, more physiological SS levels. In one intestinal chip, the highest reported SS was 0.033 dyn/cm^2^^13^, while another study investigated the effect of SS between 0–0.03 dyn/cm^2^ in the Hele–Shaw cell culture device^38^. The latter study demonstrates that the highest applied SS led to the highest levels of F-actin and microvilli, while the barrier integrity was optimal at SS values between 0.019-0.26 dyn/cm^2^. It is important to note that the study used a single-channel chip, and cells might behave differently in a dual-channel system. In the DFC model presented in this study, a robust and differentiated layer of Caco-2 cells developed and was maintained, capable of withstanding a liquid SS of at least 0.3 dyn/cm², achieved by gradually increasing the flow rate during the first seven days of culture. The continuous SS of up to 0.3 dyn/cm^2^ in the DFC likely contributed to inducing the morphology of the cultured epithelium, which often reached 50-140 µm thickness, exhibiting villus-like structures and tight junctions at 13 days of maturation. This structure was achieved without application of active mechanical stretching of the membrane as applied in some PDMS gut-on-a-chip models^13^, though the thin polyester membrane used in the DFC has some flexibility that could, during the applied sequential flow, accommodate some level of sequential stretching. It remains unclear whether membrane stretching is critical for the induction of villus-like structures in Caco-2 cells. In a study by Dogan et al., a liquid SS of up to 1.3 dyn/cm^2^ was applied to Caco-2 layers in a microtiter-well-based model without mechanical stretching^37^. This approach resulted in similar morphological features as observed in PDMS models with mechanical stretching^13^. The authors concluded that seeding density and flow rate are the two main features affecting the morphology of the cell layer^37^. Another critical aspect of SS is its influence on the induction of virulence factors in adherent bacteria. In a study by Alsharif et al., the expression of virulence factors in *E. coli* increased with increasing SS^40^. Another study by Park et al., showed that SS in the range between 0.17-0.34 dyn/cm^2^ promotes the biofilm formation of bacteria e.g. *P. aeruginosa*^41^. This, along with its role in intestinal epithelial development and maturation, highlights the importance of using physiologically correct fluid shear when studying host–microbe interactions in vitro^38^.

Because mucus is an essential part of the human intestine, the presence of both neutral and acidic mucins in the cell layer was investigated by staining with Shiff’s reagent and Alcian blue, respectively. An increasing amount of neutral mucin staining was found in the cultures during the 13 days culture period, with a higher signal in the DFC than in the static culture. The gel-forming glycoprotein MUC2 is a major component of the intestinal mucus layer—particularly in the colon—where it plays a key role in bacterial colonization^42^. Previous studies have suggested that hypoxia directly promotes MUC2 expression through HIF-1α^43^. In the DFC, anaerobic conditions led to increased MUC2 protein levels in the Caco-2 cells according to immunostaining; however, increased MUC2 could not be detected by ELISA. RNA-sequencing also indicated low basal expression levels compared to in vivo data, suggesting the levels do not reflect physiological conditions. The absence of a thick, multi-layered mucus lining above the intestinal epithelium is a known issue in Caco-2-based models. Recently, it has been shown that co-culturing the goblet cell HT29-MTX and Caco-2 produces a more sufficient mucus layer than Caco-2 cells in monoculture^44,45^. Furthermore, recent studies have demonstrated that culturing Caco-2 under air–liquid interface conditions with vasointestinal peptide leads to the formation of a functional mucus layer^46^. Similar approaches are currently being explored with our DFC model to further approach a colon-like epithelium.

Studies have shown that Caco-2 cells can differentiate into various major intestinal cell types when xenografted into mice, with indications that this differentiation also occurs when cultured in gut-mimicking microfluidic models^36,47^. To assess whether this maturation towards a more advanced intestinal epithelium occurs in our model, we used transcriptomic analysis to compare our DFC model with intestinal single-cell and bulk transcriptomic profiles. Overall, based on marker gene expression, our results align with previous studies, indicating that Caco-2 models predominantly resemble absorptive enterocytes^29^. However, our transcriptomic analysis does not resolve distinct intestinal cell subpopulations, so the presence of other cell types cannot be excluded. For example, the stronger MUC2 signal observed under anaerobic DFC conditions may suggest the presence of goblet-like phenotypes in a small subset of cells, possibly obscured in the bulk RNA-seq data. The downregulation of cilia-related pathways, combined with the upregulation of pathways associated with nutrient absorption, suggests that DFC conditions drive cells toward a more absorptive and less quiescent phenotype compared to the static model, thus more closely resembling a mature, rapidly renewing intestinal epithelium^27^. Relatively few genes were differentially expressed under anaerobic versus aerobic DFC conditions, with anaerobic conditions specifically showing upregulation of metallothionein-encoding genes and downregulation of genes involved in cell proliferation and the electron transport chain. These findings indicate that while anaerobic DFC conditions lead to some cellular adjustments compared to maintaining the DFC fully aerobic, such as reduced proliferation and hypoxic adaptation, the overall absorptive phenotype of the cells remains consistent. Interestingly, although a clear hypoxic response was evident, we observed no significant upregulation of *HIF1A* mRNA, a central mediator of hypoxia signaling. This likely reflects our sampling—taken 6 days into anaerobic culture (13 days post-seeding)—missing an earlier transient HIF1A mRNA peak that had already returned to baseline, as reported in prior studies^48,49^. Nonetheless, it remains possible that HIF1α protein is still stabilized and active under these conditions, given that its regulation is primarily post-translational during hypoxia^50^. Overall, the observation confirms that the conditions generated in the anaerobic DFC support the growth and maintenance of a viable and physiological epithelium, and provide a suitable environment for anaerobic co-culture. Comparison of the transcriptomic profile with a recently described anaerobic 3D gut model reveals strong similarities in the expression of marker genes for intestinal cell types^30^, demonstrating that a similar Caco-2 culture is achieved in our system as in more complex, PDMS-based microfluidic models. These parallels suggest a common trend toward small intestine-like characteristics in microfluidic Caco-2-based gut models, highlighting the need to also implement these models using cell lines that more closely resemble the colon, where the microbiota is concentrated and intestinal pathogens commonly manifest^3^.

*C. difficile* is a major health concern, for which in vitro models to study its interaction with the human host are critically needed^51^. *C. difficile* is an obligate anaerobic bacterium^52^ that has not previously been cultured long-term (>48 h) in an in vitro model on a viable layer of intestinal cells. Controlling the oxygen levels at the epithelium surface to permit growth of this organism is a challenging task that was made possible here with the alternative DFC design presented, combined with the online AU. To test if our model supported co-cultures of epithelial cells and *C. difficile* in the presence of a normal gut microbiota bacterium, we co-inoculated DFCs with *C. difficile* and *B. fragilis*. Subsequent treatment of simulated infection with vancomycin was performed to demonstrate the models’ applicability as a treatment test platform. Vancomycin treatment in vivo typically clears *C. difficile* symptoms, but approximately 25% of patients experience recurrent infection within three months^53^, suggesting that *C. difficile* has yet unrecognized mechanisms to survive this treatment. In the untreated control, both *B. fragilis* and *C. difficile* multiplied rapidly during the first three days of the infection (based on CFU/mL in the effluent), demonstrating that the low oxygen levels at the apical cell surface are sufficiently low to support culture of these obligate anaerobes. After this time point, bacterial shedding reached a steady state until the termination of the experiment on day five. As expected, the amount of *C. difficile* decreased following vancomycin treatment; however, vegetative bacteria were still present in the DFC after two days of treatment. Dense bacterial aggregates were observed in the vancomycin treated DFC, and while a direct protective role of these aggregates was not demonstrated in this study, the close proximity of *C. difficile* to *B. fragilis* within dense aggregates may contribute to persistence, as bacteria embedded in biofilm clumps are able to survive vancomycin far exceeding the bactericidal levels of their planktonic state^54^. Few to no planktonic *C. difficile* were found in the DFC effluent after two days of treatment with vancomycin but filamentous gram-positive rods were identified embedded in shed bacterial aggregates. Additionally, numerous bacteria were observed by TEM to be located in intracellular niches. Other pathogens, such as uropathogenic *E. coli*, are known to invade epithelial cells, creating intracellular bacterial communities that are less susceptible to antibiotics^55,56^. However, limited information is available about the intracellular lifestyle of *B. fragilis* and *C. difficile*. Recent studies have shown that *C. difficile* spores can localize to the intracellular space of intestinal epithelial cells, suggesting a potential mechanism for escaping antibiotic treatment^57^. The model presented here provides an excellent opportunity to further explore potentially invasive/intracellular lifestyles of *C. difficile* and other obligate anaerobes to enhance our understanding of the mechanisms underlying persistent and recurring intestinal infection. Although both *C. difficile* and *B. fragilis* are generally classified as obligate anaerobic bacteria, they have been shown to replicate under microoxic conditions^58,59^; however, their growth rate might be limited. While the oxygen levels achieved in the DFC fall within a physiologically relevant range^23^, tolerable for obligate anaerobic species such as *C. difficile* and *B. fragilis*, it remains unclear whether more oxygen-sensitive microbes requiring strictly anoxic conditions (<0.1%) can colonize and grow in this environment. These conditions, however, could be achieved by optimizing the length of the silicone tubing used in the AU, in conjunction with adjusting the flow rate, as illustrated in Fig. 2B.

In conclusion, we present an accessible and robust in vitro model for co-culturing of intestinal epithelial cells and obligate anaerobic bacteria. This model not only enables detailed studies of direct host–microbe interactions within a more complex, gut-mimicking, low oxygen environment but also provides a valuable platform for evaluating novel treatments for intestinal infections that may advance therapeutic strategies in the field.

## Methods

### Fabrication of the dual flow chamber

The dual flow chamber (DFC) was made by attaching two sticky-slide I Luer (Ibidi®) on both sides of a 12 µm thick tissue culture treated transparent polyester (PET) membrane with a pore size of 0.45 µm and a porosity of 0.6% (it4ip, 2000M12/640N453). To ensure a leakage tight construction, an even amount of pressure was applied to the sticky-slides using screw clamps and plastic blocks with predrilled holes for the inlet and exit for 10–15 s (see supplementary Fig. S1). Both sides of the DFC were filled with 70% ethanol and left for 20 min to sterilize followed by curing of the glue at 37 °C overnight.

### Anaerobization unit (AU)

To ensure a continuous supply of anoxic media, an ascorbate solution was made by adding 2 g of sodium L-ascorbate (Sigma-Aldrich) to 100 mL of a 0.1 M NaOH solution (Sigma-Aldrich). Inside the solution, a 150 cm long silicone tube (Helixmark®; mat.no: 456350287, inner diameter (ID): 0.99 mm, wall thickness (WT): 0.31 mm) was placed. The tube dimensions were chosen to ensure optimal exchange of oxygen. To prevent reabsorption of oxygen into the media, which happens extremely fast, the anoxic media leaving the anaerobization unit (AU) was connected to the DFC and/or oxygen sensor using stainless steel tubing. Before connecting to the DFC, culture media were flushed through the AU at a flow rate of 320 µL/min for at least one hour to stabilize the system.

### Cell culture

Caco-2 HTB-37™ cells (ATCC) were cultured in Dulbecco’s modified eagles’ media (DMEM; Gibco) supplemented with 20% heat-inactivated fetal bovine serum (HI FBS; Biowest) and 1% penicillin-streptomycin (PS; Gibco) (Stock: 10,000 units/mL Penicillin and 10,000 µg/mL Streptomycin). Cells were split when 60-90% confluent (twice–thrice weekly) and used for experiments in passage 32-54. All cultures were maintained in a standard CO_2_ incubator at 5% CO_2_ and 37 °C.

Prior to seeding of cells in DFCs and cell culture inserts (Falcon; 353090), the apical (upper) side of membranes was coated with Collagen Coating Solution (SAFC®; 125-50) by applying the solution, incubating at 37 °C for 30 min, and washing thrice with phosphate-buffered saline (PBS). In DFCs, Caco-2 cells were seeded on the membranes at a density of 1-2x10^5^ cells/cm^2^ and left to adhere for four hours before starting the flow at 15 µL/min (shear stress (SS): 0.014 dyn/cm^2^). One day post seeding (DPS) the flow was set to 60 µL/min (SS: 0.057 dyn/cm^2^) and left at this flow rate until 6 DPS. On the 6^th^ day, the flow was changed to pulsating flow (1 min flow followed by a 9-min break) with peak flow rates of 320 µL/min (SS: 0.3 dyn/cm^2^). At 7 DPS, medium was shifted to DMEM with reduced HI FBS at 2%, and flow set to a contiuous 320 µL/min (SS: 0.3 dyn/cm2) to maintain stable anaerobic conditions in the apical channel. The same flow conditions were applied at the apical and basolateral channels for all time points. Shear stress was calculated using Eq. 1 as advised by Ibidi^60^:

1 $${\rm{Shear\; stress}}\left({\rm{SS}}\right)=0,0072\times 131.6\times {\rm{flow\; rate}}({\rm{ml}}/\min )$$

For the static cultures, the cell medium was changed every third day and shifted to serum-reduced medium on day 7 post-seeding.

### Oxygen measurements

Oxygen measurements were performed using an O_2_ MicroOptode installed in a PEEK flow cell connected to a single-channel O_2_ UniAmp (UniSense, Denmark). Measurements were made every 10-60 s by flowing media through the flow cell at a flow rate between 30-1440 µL/min. Measurements were logged using SensorTrace Suite Logger software (UniSense, Denmark). The sensor was calibrated using the manufacturers’ guidelines. For oxygen measurement with the DFC, the sensor was connected to the exit site using stainless steel tubing to prevent diffusion of oxygen into the media.

The oxygen percentage at the inlet of the DFC depends on the length of the silicone coil and flow rate, thus these can be adapted to fit the desired oxygen concentration. For <0.5% oxygen at the inlet, the media must be in the silicone coil for at least 2 min. The relationship between tube length, flow rate, and achieved oxygen concentration is outlined in Fig. 2B, C. The length of the coil can then be adapted to fit the desired flow rate, by using the following formula (Eq. 2):

2 $${Lenght}({cm})=\frac{{minutes}\times {flow}({ul}/\min )}{7.38}$$

The above key is for the silicone tubing applied here and will diverge depending on silicone material and tubing dimensions.

### Immunofluorescence staining

Before staining, cells were washed with Hank’s balanced salt solution (HBSS; Gibco) thrice and fixated with a 10% neutral buffered formalin solution (Sigma-Aldrich) for 15 min. Formalin was removed and cells were washed thrice with PBS. Cells were permeabilized with 0,1% Triton X-100 in PBS for 15 min, washed thrice with PBS and blocked with 5% bovine serum albumin (BSA; Sigma-Aldrich) in PBS for 60 min. Cells were stained at room temperature with a mixture of 2 µg/mL of Occludin Monoclonal Antibody, Alexa Fluor 488 (Invitrogen; OC-3F10) and 2 µg/mL MUC2 Antibody, Alexa Fluor 405 (Novus Biologicals; 944/152) in 1% BSA in PBS for 3 h. Cells were washed with PBS and F-actin stained with 100 nM Acti-stain™ 555 Phalloidin (Cytoskeleton, Inc.) for 30 min before washing thrice with PBS. The membranes were removed from the sticky slides using scalpels and assembled in silicone frames on microscopy slides, mounted with Fluorescence Mounting Medium (Dako) and sealed with a coverslip.

### Mucus staining

The Alcian Blue/PAS staining kit (Artisan; AR16992-2) was used to visualize mucins in formalin-fixated Caco-2 cells. For staining of neutral mucins, cells were first incubated with periodic acid for 2 min, followed by washing thrice with PBS. Cells were then incubated with Shiff’s reagent for 10 min and washed with PBS thrice to remove excess staining solution. For staining of acidic mucins, cells were incubated with Alcian Blue for 15 min, followed by washing once with distilled water and trice with 3% acetic acid. Membranes were stored in PBS until imaging to prevent drying.

### Viability staining

Viability of cells was visualized using LIVE/DEAD™ Viability/Cytotoxicity Kit (Invitrogen; L3224) by adding 2 µL Calcein-AM and 2 µL Ethidium homodimer-1 to 2 mL of HBSS before transferring it to the apical channel of the DFC. Cells were stained for 30 min followed by a 15-min fixation step with formalin. The cells were washed thrice with HBSS before each step. The membranes were removed from the sticky slides using scalpels and assembled in silicone frames on microscopy slides, mounted with SlowFade^TM^ Diamond Antifade Mountant with DAPI (Invitrogen; S36968) and sealed with a coverslip.

### RNA isolation

To isolate RNA from Caco-2 cells grown in DFCs, culture medium was aspirated, and ice-cold lysis buffer (4 M guanidinium thiocyanate (GITC), 0.02 mM Tris-HCl (pH 7.5), 10 mM NaAcetate pH 4.5, 25 mM EDTA, 0.1% Triton X-100, 2 mM DTT) was added. The cells were sheared from the DFC by rapidly moving a syringe, fitted into the Luer port of the apical channel outlet, up and down. The lysate was then transferred to RNase-free 1.5 mL tubes and snap-frozen in liquid nitrogen. RNA extraction was subsequently performed using a phenol-chloroform method. Briefly, 300 µL of lysate was mixed with 150 μL of solution 2 (10 mM Na-acetate pH 4.5, and 2% SDS), 700 μL of acidic phenol (pH 4.5) and 300 μL of chloroform. Tubes were inverted and heated at 80 °C for 3-4 min, then cooled on ice. After centrifugation at 10,000*g* for 5 min, the aqueous phase was transferred to 96% ethanol with Na-acetate (37.5 mM) and precipitated overnight (ON). RNA was pelleted by centrifugation (20,000*g* for 45 min), washed in ice-cold ethanol, resuspended in RNase-free H_2_O, and stored at −20 °C or −80 °C.

### RNA-sequencing

RNA samples from Caco-2 cultures grown in DFCs were depleted of ribosomal RNA using the NEBNext rRNA Depletion Kit (Human/Mouse/Rat). RNA sequencing library preparation was performed with the NEBNext Ultra II Directional RNA Library Prep Kit for Illumina (New England Biolabs) and paired-end sequencing was conducted on a NovaSeq 6000 System (Illumina). Raw paired-end reads were aligned to the human genome assembly GRCh38/hg38 using STAR (version 2.7.11a). Primary alignments were filtered, sorted, and indexed using samtools (version 1.19). For feature counting, only protein-coding genes were considered. Read counts within exons were determined using featureCounts from the subread package (version 2.0.6), with multimapping reads included and counted fractionally. Differential gene expression analysis was conducted with edgeR (version 4.2.1), identifying genes with |log2(FC)| > 2 and FDR < 0.05 as significantly differentially expressed. Pathway enrichment on significant DEGs was conducted using the webtool MetaScape with Reactome gene sets^25^. For marker gene expression analysis, lineage marker genes from Burclaff et al.^61^ were used. Normalized gene expression data (normalized transcript per million, nTPM) from the “RNA single cell type data” and “RNA consensus tissue gene data” datasets from The Human Protein Atlas were used for comparison^62,63^. Clustered heatmaps were generated with the R package pheatmap (version 1.0.12), using Ward’s method (“ward.D2”) for hierarchical clustering.

### Colonization with *Clostridioides difficile* and *Bacteroides fragilis*

Caco-2 cells were matured aerobically for seven days in the DFC, as previously described. On the day of inoculation, the media were replaced with DMEM containing 2% HI FBS, and the AU was connected to the inlet of the apical channel. Antibiotic-free media was flushed through both channels of the DFC for one hour before infection. Bacterial suspensions of *C. difficile* (ATCC 700057) and *B. fragilis* (ATCC 25285), grown anaerobically overnight on 5% blood agar plates, were adjusted to an optical density at 600 nm (OD600) of 0.2 in deoxygenated Hanks’ Balanced Salt Solution (HBSS, Gibco). A mixture of *B. fragilis* and *C. difficile* was made from the solutions (OD ratio 1:9) and introduced through the apical channel at a flow rate of 320 µl/min until the suspension reached the top of the outlet reservoir. The colony forming unit (CFU) per mL in the inoculum was 5*10^6^ for *C. difficile* and 1*10^8^ for *B. fragilis*. This ratio was selected to promote co-colonization by both bacterial species while reflecting a predominance of the commensal microbiota, as represented by *B. fragilis*. The discrepancy between CFU counts can be explained by the difference in optical absorption, as *C. difficile* CFU are >100 times lower than *B. fragilis*, when adjusting to the same OD600. The flow was then stopped for 10 min to allow for initial bacterial attachment, after which the flow rate was resumed at 320 µl/min. Daily effluent samples from the apical channel were plated on selective agar plates (CHROMID® *C. difficile* agar plates and brain heart infusion agar plates with 6 µg/mL vancomycin (Bactocin®, MIP Pharma)) to measure CFU. On day 3, the medium in DFCs allocated for antibiotic treatment was changed to DMEM with 2% HI FBS containing 6 µg/mL vancomycin. On day 5, loose aggregates were aspirated, and the cell layers of the DFCs were harvested using Trypsin-EDTA (0.25%; Biowest) with 0.1% Triton X-100, then plated for CFU enumeration. Gram staining was performed on loose aggregates harvested from the apical channels. For microscopy (confocal laser scanning microscopy (CLSM) or transmission electron microscopy (TEM)), the infected cell layers were not harvested but instead prepared according to the appropriate protocol.

### Microscopy

Live cultures of Caco-2 cells were imaged using an Olympus CKX53 inverted microscope connected to an Olympus SC50 camera and using Olympus cellSens software. PAS-stained cultures were imaged using a Leica DM4 B microscope using LAS X software. Cells were imaged in the middle of the flow chamber. Gram-stained aggregates harvested from the apical channel of the flow chambers were imaged using a Leica DM3000 LED microscope connected to a Flexacam C1 and using LAS X software. Immunofluorescence stained and LIVE/DEAD stained cultures were imaged using either a Nikon AX or an Olympus FV1000 MPE CLSM. Cultures were imaged between the inlet and the center of the flow chamber.

### Transmission electron microscopy

The Caco-2 cells were matured in flow chambers for seven days followed by inoculation with *C. difficile* and *B. fragilis* and treatment with vancomycin as described previously. The cell cultures were fixed using a 2% glutaraldehyde solution in 0.04 M phosphate buffer. The membranes with adhering cell cultures were cut out from the flow chambers, washed with 0.1 M phosphate buffer, and stained with 1% osmium for 90 min. Following staining, the membranes were washed in phosphate buffer and water, serially dehydrated in ethanol and acetone, and infiltrated with TAAB 812 Embedding Resin (T030, TAAB).

Ultrathin (60 nm) sections were cut on a Leica Ultracut UCT microtome. The sections were collected on Formvar support film copper grids (FF2010-CU-50, Electron Microscopy Sciences). The grids were stained with 3% uranyl acetate for 15 min. at 60 °C and 3% lead citrate (Leica Ultrostain 2) for 6 min at room temperature. The cells were photographed using a JEM-1400 Plus transmission electron microscope, equipped with Quemsa TEM CCD camera and Radius imaging software.

### Statistics

Statistical analyses were performed using R (version 4.3.2). Normality of the differences in paired oxygen concentration measurements was assessed using the Shapiro-Wilk test. Based on this, a paired t-test was conducted using the compare_means function from the ggpubr package (method = “t.test”). For the comparison of CFUs between treated and untreated DFCs, a non-parametric test (Wilcoxon rank-sum test) (method = “wilcox.test”) was applied.

### Resource availability

#### Lead contact

For further information, professor Thomas Emil Andersen (thandersen@health.sdu.dk) can be contacted.

## Supplementary information

Supplementary Materials

## Data Availability

The RNA-sequencing data discussed in this publication have been deposited in NCBI’s Gene Expression Omnibus^64^ and are accessible through GEO Series accession number GSE284670 (https://www.ncbi.nlm.nih.gov/geo/query/acc.cgi?acc= GSE284670).

## Code availability

The RNA-sequencing data discussed in this publication have been deposited in NCBI’s Gene Expression Omnibus^64^ and are accessible through GEO Series accession number GSE284670 (https://www.ncbi.nlm.nih.gov/geo/query/acc.cgi?acc= GSE284670).
