# Supplementary material for: An anaerobic in vitro flow model for studying interactions at the gastrointestinal host–microbe interface
Source: NPJ Biofilms Microbiomes. 2025 Aug 11;11:160. doi: 10.1038/s41522-025-00800-z (PMC12340051; doi:10.1038/s41522-025-00800-z)
Supplement: Supplementary file 1 — Supplementary Materials [file 41522_2025_800_MOESM1_ESM.docx]

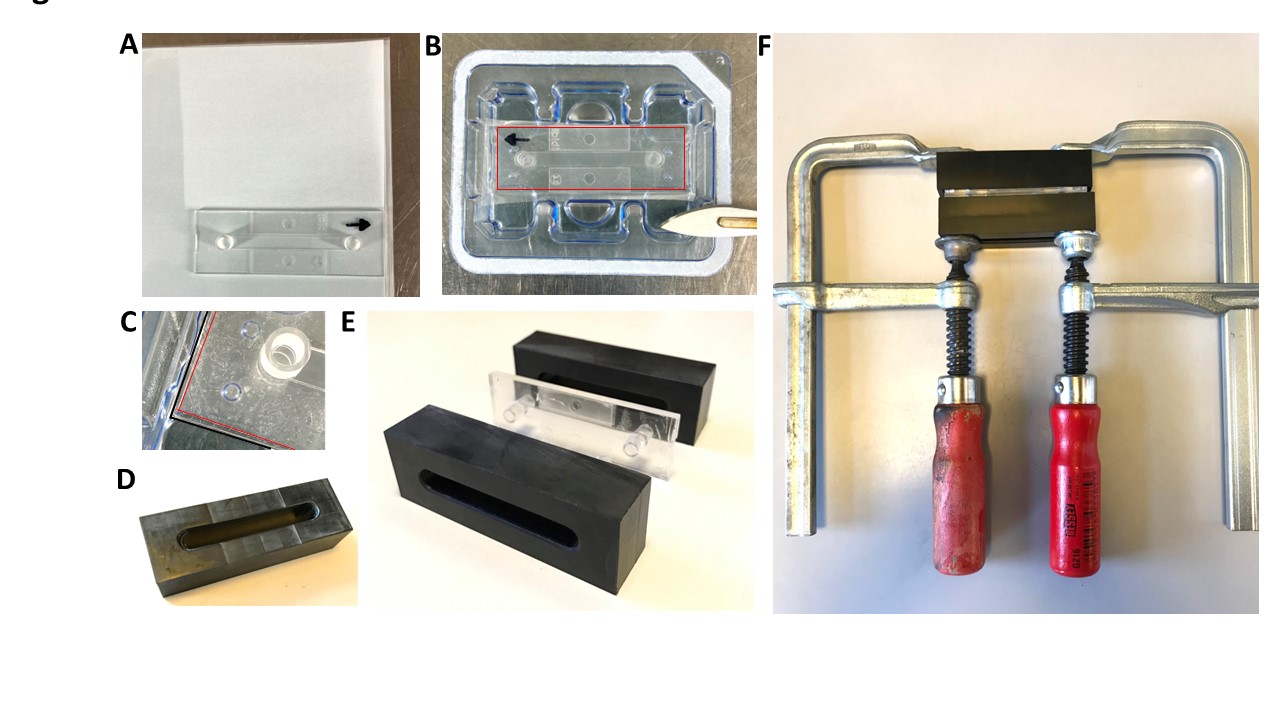
**Supplementary figures**

**Supplementary figure S1**: **Assembly of the double flow chamber.** One sticky slide I luer 0.4 mm (Ibidi) is placed on top of the 12 µm thin polyester (PET) membrane (pore size: 0.4 µm, it4ip) and tightly secured by pulling and stretching while applying a light pressure to the sides (**A**). Then the edge of the membrane is cut approximately 1mm inside the sticky slide (indicated by the red line) (**B, C**) followed by placing of another sticky slide to the other side of the membrane. The two sticky slides are pressed together using to plastic blocks and screw clamps, to ensure removal of air pockets between the membrane and sticky slides (**D, E, F**).


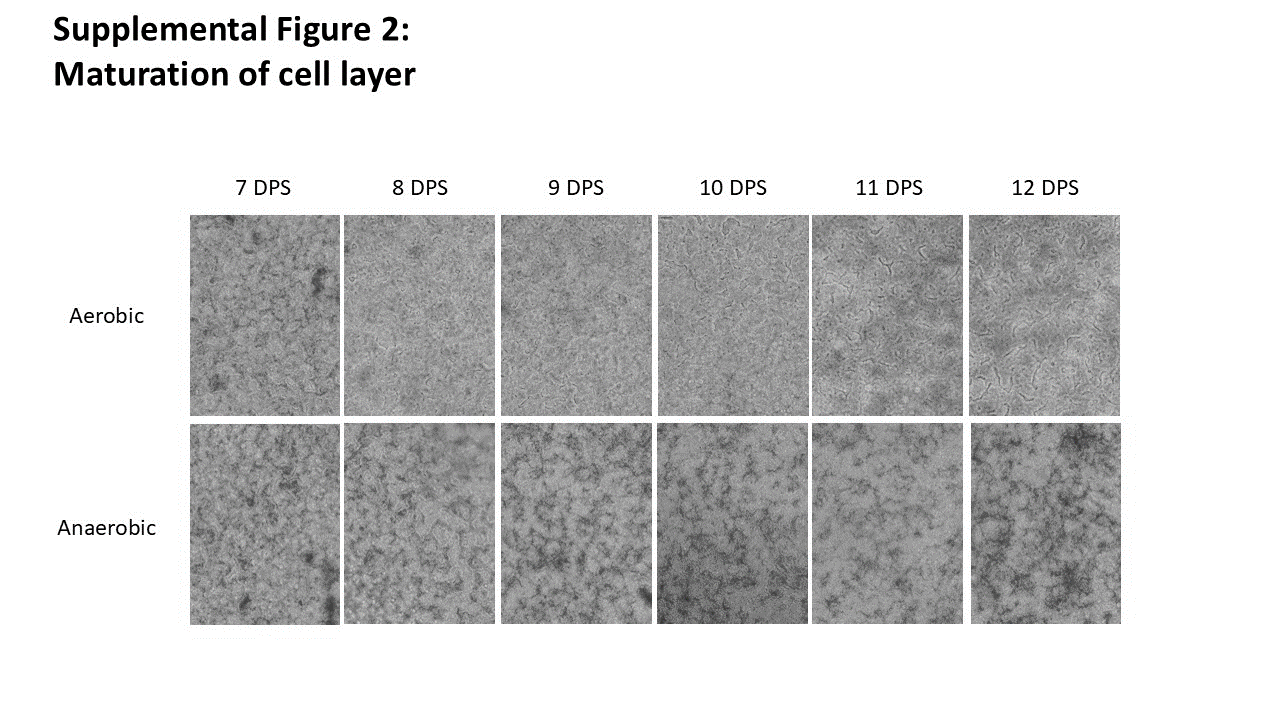


**Supplementary figure S2**: **Maturation of Caco-2 cells during aerobic and anaerobic conditions**. Brightfield images of Caco-2 cells 7-12 days post seeding (DPS). Cells cultured in the oxygen saturated DFC (aerobic row) show high villus-like structures that continues to grow during the period. Cells cultured in the anaerobic DFC pulls towards the polyester membrane.


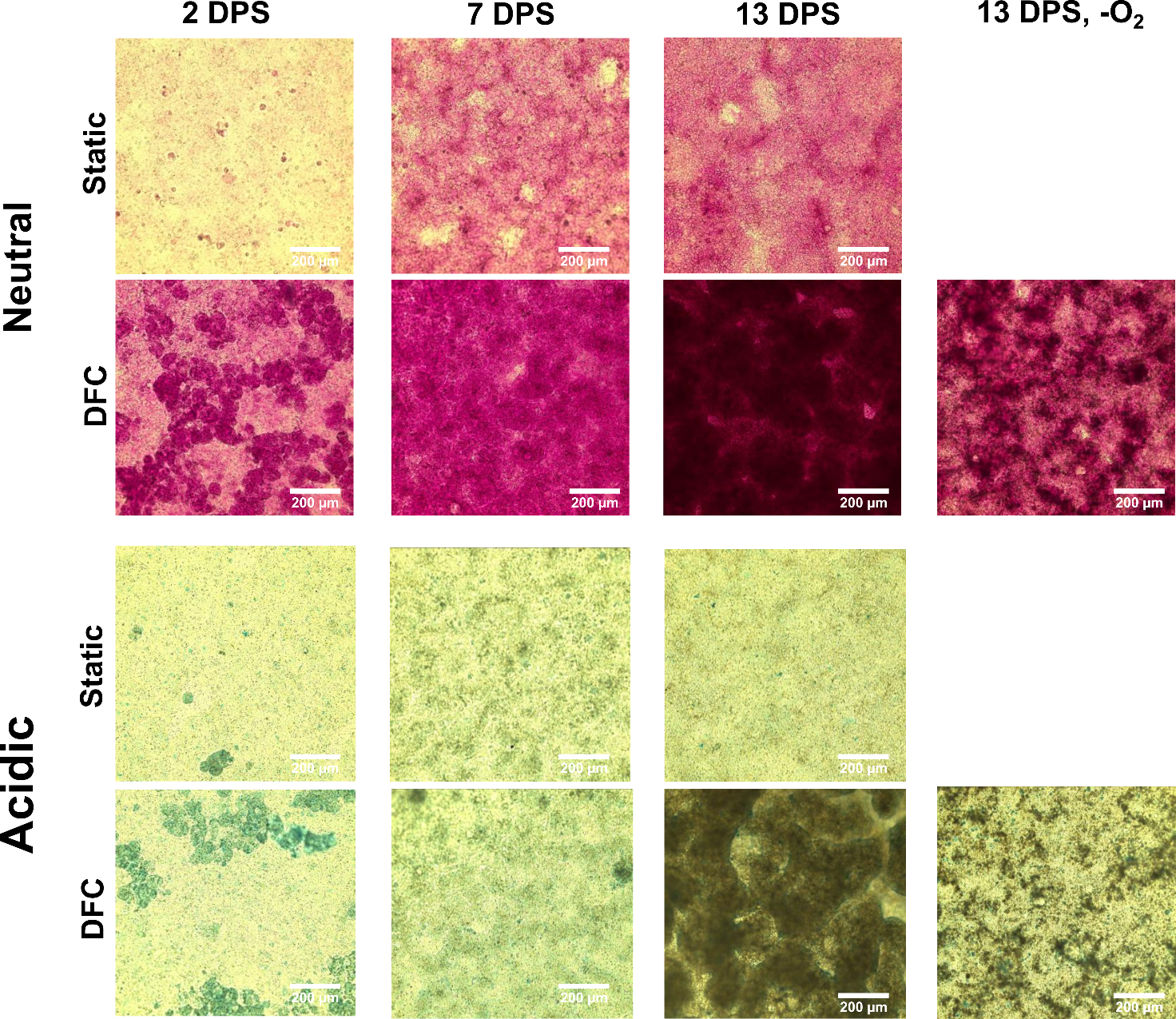


**Supplementary figure S3**: **Staining of neutral (Shiff’s reagent) and acidic (Alcian blue) mucins.** Caco-2 cells were grown for 2-13 days post seeding (DPS) in cell culture inserts (static) and in the double flow chambers (DFCs) under both aerobic (2DPS, 7DPS, 13DPS) and anaerobic (13DPS, -O_2_) conditions in the apical channel. Cells were fixated with formalin at different time points and stained for neutral and acidic mucins (see main manuscript for materials and methods).


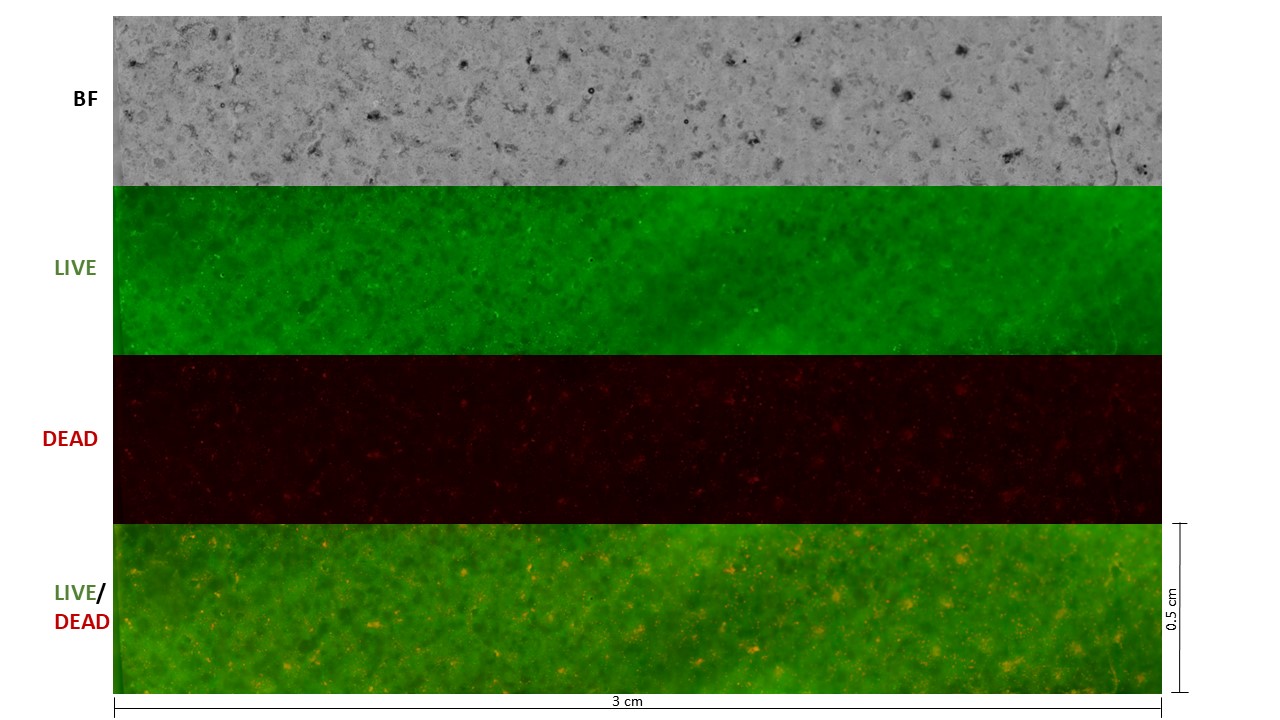


**Supplementary figure S4**: **Viability of Caco-2 cells after 21 days of anaerobic culturing.** Caco-2 cells were matured aerobically for 7 days in double flow chambers (DFCs) before shifting to anaerobic media in the apical channel. The antioxidant (ascorbate) solution of the anaerobizer unit was changed every seventh day. After 21 days of anaerobic culturing, cells were stained for live (green) and dead (red) cells using LIVE/DEAD™ Viability/Cytotoxicity Kit (Invitrogen; L3224). Stitched images were made using the multiple image alignment feature of Olympus cellSens® software and displays the entire with of the chamber channel and 3/5 of the channel length. At the time of termination oxygen levels in the efflux from the apical channel were measured to be below 0.5%.


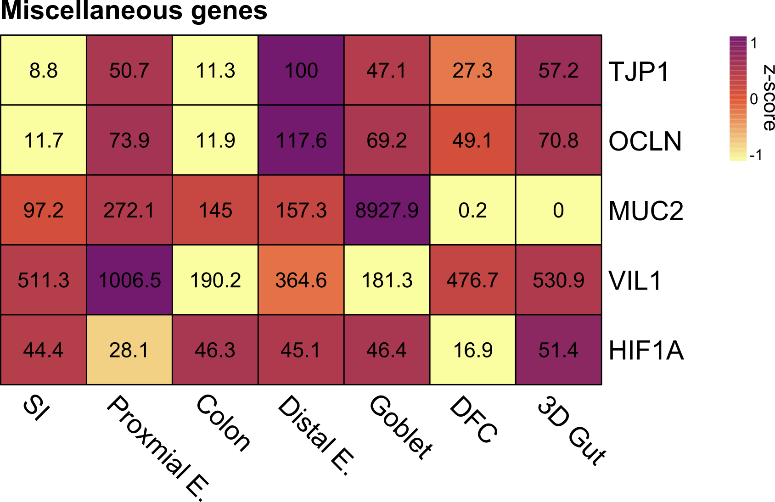


**Supplementary figure S5: Heatmap of miscellaneous gene expression in human intestinal tissues, cell types and gut models.** Normalized TPM (nTPM) values for five genes (TJP1, OCLN, MUC2, VIL1, and HIF1A)—obtained by applying TMM normalization factors (edgeR) to raw TPMs—are shown across seven sample types: human small intestine (SI) and colon tissues (derived from bulk RNA sequencing), as well as proximal and distal enterocytes, and goblet cells (derived from single-cell RNA sequencing), as well as an anaerobic intestinal chip model (3D Gut), and our own anaerobic dual flow chamber (DFC) model at 13 days post seeding. Rows are scaled (z-score), and overlaid numbers report the original nTPM values.


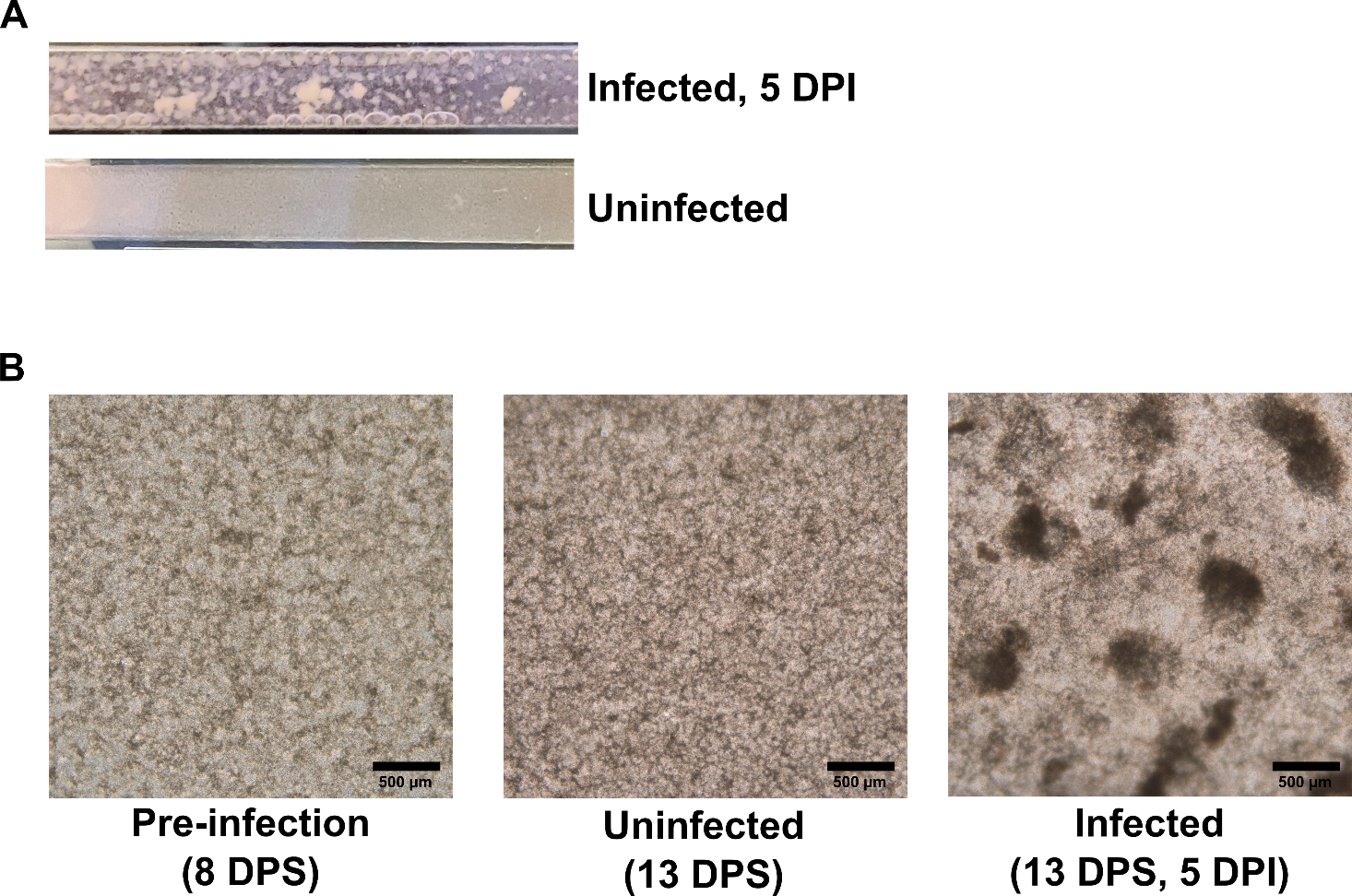


**Supplementary figure S6: Visible macrocolonies present on Caco-2 cell layers at 5 days post infection in the dual flow chamber model.** At eight days post seeding (DPS), Caco-2 cells were infected with *B. fragilis* and *C. difficile*. At five days post infection (DPI), macrocolonies were visible by the naked eye (A) and under light microscopy (B) throughout the chamber.
